# Can 3D Printing Bring Droplet Microfluidics to Every Lab?—A Systematic Review

**DOI:** 10.3390/mi12030339

**Published:** 2021-03-22

**Authors:** Nafisat Gyimah, Ott Scheler, Toomas Rang, Tamas Pardy

**Affiliations:** 1 Thomas Johann Seebeck Department of Electronics, Tallinn University of Technology, 19086 Tallinn, Estonia; nafisat.gyimah@taltech.ee (N.G.); toomas.rang@taltech.ee (T.R.); 2 Department of Chemistry and Biotechnology, Tallinn University of Technology, 19086 Tallinn, Estonia; ott.scheler@taltech.ee

**Keywords:** 3D printing, microfluidics, automation, lab-on-a-chip, open-source hardware

## Abstract

In recent years, additive manufacturing has steadily gained attention in both research and industry. Applications range from prototyping to small-scale production, with 3D printing offering reduced logistics overheads, better design flexibility and ease of use compared with traditional fabrication methods. In addition, printer and material costs have also decreased rapidly. These advantages make 3D printing attractive for application in microfluidic chip fabrication. However, 3D printing microfluidics is still a new area. Is the technology mature enough to print complex microchannel geometries, such as droplet microfluidics? Can 3D-printed droplet microfluidic chips be used in biological or chemical applications? Is 3D printing mature enough to be used in every research lab? These are the questions we will seek answers to in our systematic review. We will analyze (1) the key performance metrics of 3D-printed droplet microfluidics and (2) existing biological or chemical application areas. In addition, we evaluate (3) the potential of large-scale application of 3D printing microfluidics. Finally, (4) we discuss how 3D printing and digital design automation could trivialize microfluidic chip fabrication in the long term. Based on our analysis, we can conclude that today, 3D printers could already be used in every research lab. Printing droplet microfluidics is also a possibility, albeit with some challenges discussed in this review.

## 1. Introduction

Additive manufacturing of microfluidics has recently gained in popularity, and increasingly complex 3D-printed (3DP) microfluidic chips are demonstrated in the literature [1,2,3,4,5,6,7,8,9,10,11,12,13,14,15]. At the same time, printers are becoming cheaper and simpler to use, offering far wider availability than before [1,11]. Printing materials have also improved, and more recent options are significantly better at biological and chemical compatibility [2,14,16,17]. This trend enables a growing number of biological and chemical applications [6,18,19,20,21,22]. Is it perhaps the right time for every research lab to own a 3D printer?

In this paper, we seek answers to whether and how 3DP microfluidics could be applied in mainstream laboratories. We analyze this problem through an increasingly popular but highly complex application area: 3DP droplet microfluidics. Droplet microfluidics, as opposed to continuous flow microfluidics, is performed in discrete fluid droplets. Commonly, this is realized by generating two-phase flows of immiscible phases, such as aqueous droplets flowing in oil [23]. Droplet microfluidics enables the manipulation of small sample and reagent volumes in physically discrete droplets. This in turn allows high-throughput analysis without losing sensitivity [24]. Thus, the technology has recently emerged as a tool in microbiology for imaging analysis of large cell populations [25,26]. In addition, droplet microfluidics has applications in nanotechnology [27]. Recently, applications in nucleic acid analysis have also been demonstrated [28]. Although the paper will focus on 3DP droplet microfluidics in detail, the problems discussed in the paper generally apply to all 3DP microfluidics, and in some cases, a wider scope was needed for the analysis, so we selectively included general 3DP microfluidics in the review.

**Figure 1:**
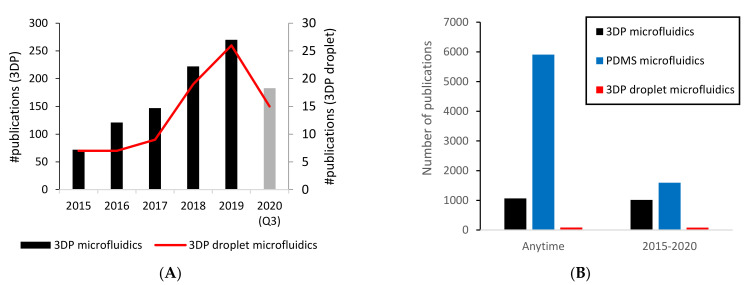
The number of scientific papers about 3D-printed (3DP) microfluidics has increased nearly fourfold over the last five years. Based on the number of publications reported by Web of Science, (**A**) interest toward 3DP microfluidics has steadily risen during the last five years. Droplet microfluidics is a fairly new application area for 3DP microfluidics that has rapidly risen in popularity since 2015. Furthermore, (**B**) while polydimethylsiloxane (PDMS) is still the primary choice of material for microfluidic chips, new publications have been in a rapid decline. In comparison, most 3DP microfluidics papers have been published since 2015. Search parameters and results are listed in Table S1 in supplementary material S1.

First, we evaluated the popularity of 3DP microfluidics and 3DP droplet microfluidics in recent years, based on the number of publications. We also compared the popularity of 3DP microfluidics and polydimethylsiloxane (PDMS) microfluidics. Our evaluation is based on Web of Science, and all search parameters and results can be found in Table S1 in supplementary material S1. As shown in Figure 1A, the number of publications in 3DP microfluidics has increased close to four times in the last five years. The trend is similar with 3DP droplet microfluidics, although the number of papers is still far smaller (Figure 1A). This smaller number only indicates that 3DP droplet microfluidics is a new and upcoming field within 3DP microfluidics that is rapidly gaining interest. It is also very clear that PDMS-based microfluidics is by far the most popular option for chip fabrication among researchers (Figure 1B). This is evidenced by the close to five times difference in the number of publications compared with 3DP microfluidics when we search without a time limit. However, if we search only within the last five years, it becomes clear that the 3DP microfluidics field has grown far more rapidly than PDMS-based microfluidics (Figure 1B). In addition, 3DP droplet microfluidics papers are almost without exception from the last five years. Why does this trend exist? We will analyze and point out possible reasons in the second half of this review.

Second, we evaluated review papers from the last five years on 3DP microfluidics and 3DP droplet microfluidics. The review papers are categorized in Table 1. The first level of grouping is based on the features discussed in the reviews, namely the following:

- Technology, referring to reviews on 3DP technology as it relates to microfluidics, challenges, limitations and comparisons of options, among other factors;
- Applications, be they biological or chemical, as well as the droplet microfluidics applications of 3DP microfluidic chips;
- Future potential, namely novel technology options that 3DP technology can offer (e.g., cloud-based manufacturing, digital design automation, as well as the potential of constructing integrated 3DP microfluidic systems (system integration));
- Other, which are papers that did not fit in the above categories.

The second level of grouping is based on specific topics discussed in the papers, such as cell culturing or integration with 3DP electronics. Please note that review papers can fall into more than one category in both levels of grouping. Finally, papers are grouped based on how extensive their coverage of a particular topic is. They are rated from one to three stars, where one star refers to papers with at least one paragraph on a particular topic (<1 page), two stars means at least one full section (approximately 1–3 pages) and three starts means more than one section (>3 pages).

Based on this classification, it is clear that a large number of papers have discussed 3DP technology, materials and chip printing. This is by far the most extensively covered area in reviews, and several well-illustrated descriptions of the various 3DP technologies are available. Additionally, technical challenges and limitations related to 3DP microfluidics are discussed in sufficient detail. This is not surprising, considering the novelty in 3D printing during the last five years. What is surprising, however, is the number of review papers (as well as technical reports) on the chemical or biological applications of 3DP microfluidics. The number of papers on base technology could give the wrong impression that the field is still in its infancy. It is not, as evidenced by the number of demonstrated applications in chemistry and biology, including such complex applications as cell culturing and organ-on-chip [6,18,19,20,21,22]. In addition, a significant number of reviews have analyzed biological and chemical compatibility [2,14,16,17]. Droplet microfluidics is not covered nearly as well in reviews. Furthermore, the future potential of 3DP microfluidics is largely unexplored. In addition, quantitative analysis of 3DP microfluidics with a statistical approach is very rare.

Therefore, we intend to take a more systematic and quantitative approach than previous reviews, focus on droplet microfluidics and analyze the future potential lying in 3DP microfluidics. We will analyze how digital design automation, a natural combination with 3DP technology, could change the field of microfluidic chip fabrication. The review will be divided into two main sections. In Section 2, we analyze the performance of 3DP droplet microfluidics, and in Section 3, we evaluate the potential to apply 3DP droplet microfluidics on a larger scale and the advantages that could come with it. In addition, in Section 2.3, we discuss the biological and chemical applications of 3DP droplet microfluidics. In Section 3, we analyze digital design automation and digital twins—natural pairings with 3DP technology—and how they could speed up the fabrication of microfluidics as well as decrease associated costs. Based on this analysis, we will finally analyze how 3DP microfluidics could supplement or replace more traditional fabrication techniques (e.g., PDMS soft lithography) on a larger scale.

## 2. 3D-Printed Droplet Microfluidics

In this section, we analyze whether and how 3D printing can be applied to the fabrication of droplet microfluidic chips. Given the geometrical complexity of these chips, this analysis will highlight the opportunities and challenges related to large-scale fabrication via 3D printing.

First, we focus on the key performance metrics of 3D printing technologies. Second, we analyze the relation between the channel dimensions and droplet generation parameters to find out how well 3DP droplet generators can work. Third, we analyze the biological and chemical applicability of 3DP droplet chips. This will reveal how well these chips can be applied in diagnostic and analytical settings and, therefore, how viable they can be in large-scale applications. There is a considerably lower number of papers specifically discussing 3DP droplet microfluidics compared with 3DP microfluidics in general. Therefore, we will discuss 3DP droplet microfluidics as a subset of 3DP microfluidics and indicate in the text where the analysis is limited to 3DP droplet microfluidics specifically.

### 2.1. 3D Printing Techniques

To begin our analysis, we will briefly summarize the key performance metrics of 3D printing methods as well as compare their relative popularity. Since several highly detailed overviews exist on the forms of 3D printing technology themselves [1,2,3,4,5,6,7,8,9,10,11,12,13,14,15], we will not discuss that aspect in detail. In addition, a detailed review exists on channel layouts [31], so we will instead focus on the quantitative analysis of channel geometries, which has so far been not covered in other works.

**Figure 2:**
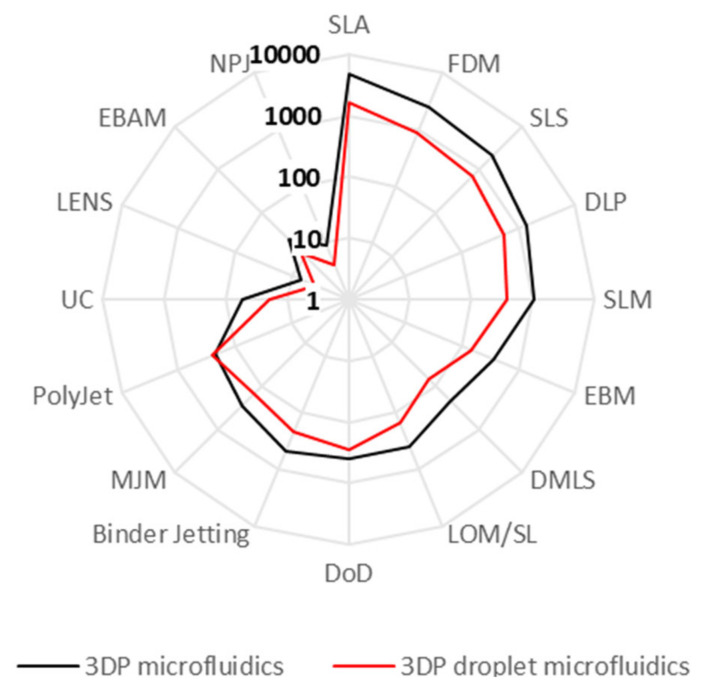
In the last five years, stereolithography has been by far the most popular 3D printing method for microfluidics. Based on the number of publications in the index of Google Scholar from the last five years, stereolithography (SLA) has been by far the most popular printing method for 3DP microfluidics, followed by fused deposition modeling (FDM), selective laser sintering (SLS) and digital light projection (DLP). However, from the 106 papers analyzed in this review, none used SLS for printing channels. Likely, SLS was used for other purposes, such as printing supporting infrastructure or tissue scaffolds connected to microfluidic systems. Abbreviations of all the listed 3DP methods are defined in Table S3 in supplementary material S3.

First, we will look at the relative popularity of different 3D printing techniques to find out which is most applicable to printing droplet generators. The index built by Google Scholar was chosen for this assessment due to its wider coverage compared with other indexers, as well as in-text search options. The search terms and detailed search results are available in Table S1 in supplementary material S1. The results are summarized in Figure 2. In addition, Table 2 summarizes the key performance parameters associated with these printing techniques (typical and best channel dimensions, printing times and average chip cost). Tables S4 and S5 in supplementary material S3 provide examples and information on 3DP materials and printers (e.g., best resolution and cost).

In terms of vat polymerization or resin 3D printing, stereolithography (SLA) and digital light projection (DLP) are similar techniques. They both operate based on selective curing of a photosensitive polymer to create voxels. However, while SLA uses a laser or a UV LCD or LED screen as the light source, DLP printers use a mercury lamp and a digital micromirror device to guide the light. By the number of indexed publications, SLA is by far the most popular technique for the 3D printing of microfluidics in general, as well as droplet microfluidics in particular. This is likely due to the fact that SLA is fairly cheap and easy to use, can implement overhangs (and therefore channels), produces relatively smooth surfaces and has a good resolution. In one example, channel dimensions down to 18 µm were demonstrated [38]. Chip costs are generally below EUR 1, but with size optimization this can go down to ~EUR 0.5, which makes the technique suitable for large-scale printing [49,50,51,52,53]. In addition, the printing time can be as low as 0.5 h [40]. Furthermore, with SLA, translucent chips can be printed [51,54,55,56,57,58,59,60,61,62,63,64,65,66,67,68,69,70]. While similar in its method, DLP is more complex technologically and therefore typically more expensive than cheaper SLA options. At the same time, SLA offers better performance parameters for microfluidic applications. In addition, cheap SLA printers (e.g., the Anycubic Photon Zero [71]) are available under EUR 200 and employ a simple UV LCD screen. The screen projects images of the layers into the resin to selectively cure it. This makes SLA an excellent candidate for the large-scale application of 3D printing microfluidic chips. The resin costs are also reasonable, and as the technology spreads, the situation will only improve. However, resin has biochemical compatibility issues, as discussed in numerous works [6,18,19,20,21,22].

Fused deposition modeling (FDM) is the second-most popular technique, likely due to its availability and low cost, as well as the wide material selection and biocompatibility of the materials. In terms of the material, FDM would be an ideal candidate, but watertight microfluidic chips are difficult to print with FDM [51,56,58,67]. The resolution is typically worse than with SLA, and the fibers are often packed loosely enough to let water through. Furthermore, proper microfluidic chips can only be printed with multi-material printers, as regular FDM does not support overhangs (i.e., channels would collapse). Therefore, typically, a water-soluble support is used, which increases printing costs and the cost of the printer. Despite all these challenges, channel dimensions as low as 40 µm have been reported [41]. Even though clear filament is available, printing translucent chips with FDM is challenging due to the misalignment of filament threads. To improve the surface quality, FDM chips could be post-processed by being polished chemically or mechanically [72]. FDM is generally the cheapest and fastest option for printing chips in bulk, so if technical challenges are addressed, it could be an ideal candidate for large-scale automated fabrication.

Material jetting methods (MultiJet printing (MJP), MultiJet monitoring (MJM) and PolyJet) can produce an excellent surface finish, as well as highly transparent parts [43,44,73,74,75,76,77,78,79]. Jetted materials are also more biocompatible than SLA parts, and the resolution can be on par with SLA. However, the printer costs are over EUR 50,000, which is far more expensive than SLA and FDM printers. The per part costs are also above EUR 1, which is significantly higher than with SLA and FDM.

While SLS and selective laser melting (SLM) are highly popular in biomedical engineering (e.g., for metal prosthetics and implants) [2], they are more difficult to apply to printing microfluidic channels. None of the 106 papers analyzed in this review used SLS or SLM for printing microfluidic channels, likely due to the fact that sintered structures tend to be porous. SLS or SLM could, however, be used for printing tissue scaffolds [2].

In summation, SLA and FDM are the best candidates for large-scale fabrication of 3DP microfluidics. Both offer the same high level of automation and flexibility. However, both have their unique technical challenges. SLA parts have issues with biocompatibility, and FDM has issues with surface quality, being watertight and printing overhangs. However, recently it has been demonstrated that some SLA materials (e.g., VisiJet SL Clear) are very close to PDMS in biocompatibility [54]. With a rising market demand and targeted optimization of resin compositions, this challenge will likely be overcome soon. Therefore, SLA is the most likely candidate for large-scale chip fabrication from a technological perspective.

### 2.2. Droplet Generation Performance of 3D-Printed Droplet Microfluidics

We continue our analysis by directly looking at the droplet generation performance of 3DP chips. We will analyze the relation between the geometry and droplet generation parameters to find out whether 3DP microfluidics can be adequate for droplet generation purposes. To determine the target ranges for our analysis, we will use PDMS chips as the reference. They represent the state of the art both in terms of popularity and performance. The typical PDMS-based droplet microfluidic chip can generate droplets at a ~100–1000 Hz generation rate in a 10–200 µm droplet diameter range with high monodispersity (CV <2%) [80,81,82,83]. We analyzed 106 recent papers, whose detailed data are available in supplementary material S2. For the detailed analysis of performance metrics and dimensional data, a dataset was composed from these 106 papers. Numeric data were collected and organized in Table S2. If necessary, unit conversions were made. Then, the data were plotted as described and shown in Figures 4–6. Data analysis was done based on the plots.

**Figure 3:**
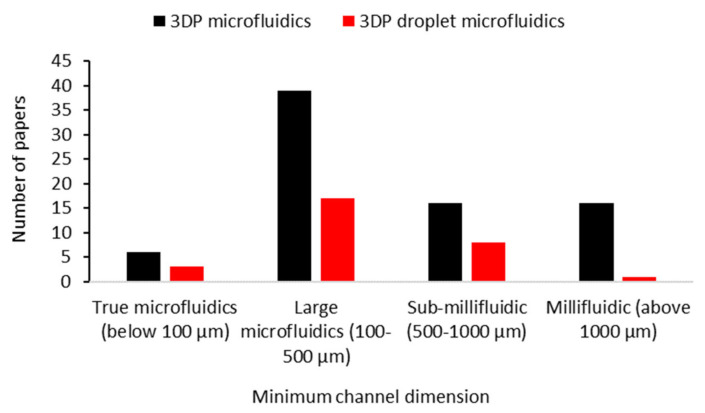
3DP microfluidic chips usually have channels within the large microfluidics size domain of 100–500 µm. Surveying the minimum channel dimensions reported in papers over the last five years revealed that most 3DP microfluidics, including droplet microfluidics, were in the category of large microfluidics with 100–500 µm channel dimensions. As evidenced by our comparison in Figure 7, this dimension range is adequate for various biological and chemical assays. Only a few examples were in the true microfluidics group of <100 µm channel dimensions, which indicates that while the technology is capable of producing smaller feature sizes, most biological and chemical applications do not need it. The size domains and their names were based on [12,41].

First of all, surveying the distribution of channel dimensions in papers (Figure 3), it is clear that most chips have channels within the 100–500 µm range. This is the range which is comfortably printable on most SLA printers [40,52,53,60,65,84,85,86,87,88,89,90,91,92,93,94,95,96,97,98,99,100], but it is also achievable with material jetting and, after some fine-tuning, with FDM [42,101,102,103,104,105,106,107]. This size regime is still within the microfluidics domain, although larger than mainstream microfluidic channels.

**Figure 4:**
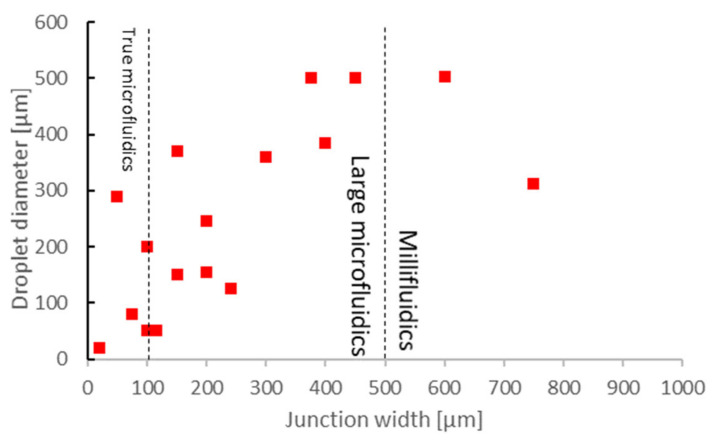
In droplet microfluidics, 3DP microfluidic chips have been mostly used in a 100–500 µm junction width regime (large microfluidics). The droplet diameter changes close to linearly with the junction width, typically also in the 100–500 µm range, although some variations are possible due to different reagents and flow rates used. By using a wider outlet and a lower flow rate, the droplet diameters can be significantly larger than the junction width. Conversely, a high flow rate can result in smaller droplets than the junction width.

However, is this adequate for droplet generation purposes? To answer this question, we must analyze the relation between channel dimensions and droplet generation metrics, such as the diameter, generation rate and monodispersity. Monodispersity is defined as the coefficient of variation of the droplet size. Figure 4 shows the relation between the junction width and droplet diameters. As stated before, most of the 106 surveyed papers reported chips in the 100–500 µm range. The monodispersity was typically 5–6% (Figure 6), and the droplet diameters were also between 50–500 µm [45,79,91,94,102,107,108,109,110,111,112]. Both parameters depended on the flow rates and reagents used, as well as the outlet width. However, we can safely conclude that functional droplet generators can be 3DP in the large microfluidics size domain of 100–500 µm. Close to half of the chips with 5–6% monodispersity were printed with SLA, again emphasizing the viability of SLA-printed chips [79,94,107,111,112].

**Figure 5:**
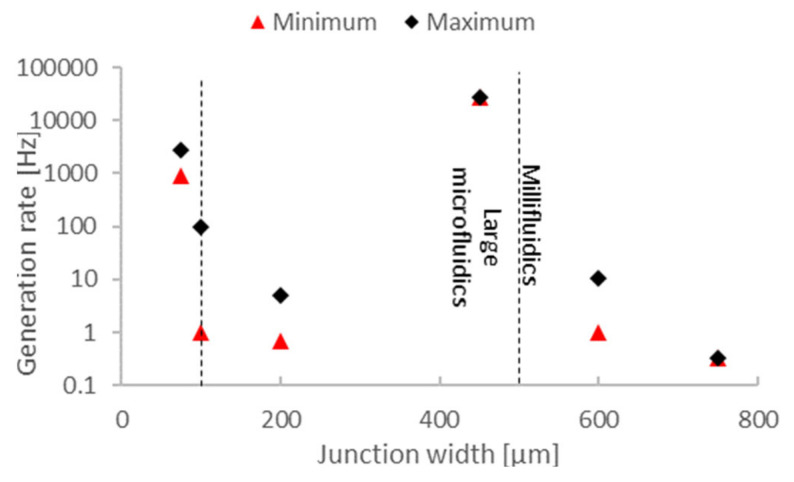
The generation rate of 3DP droplet microfluidic chips varies from 1 Hz up to 10 kHz in range. Comparing the junction width and the reported droplet generation rates, we can see that 3DP droplet generators are capable of reliably generating droplets at rates <100 Hz. Some demonstrated applications exist that have exceeded a 1 kHz generation rate, but the majority of 3DP droplet microfluidics with junctions between 100 and 500 µm in width could generate droplets at up to a 100 Hz generation rate. However, due to the scarcity of data, the relationship between the channel geometry and generation rate should be analyzed further.

**Figure 6:**
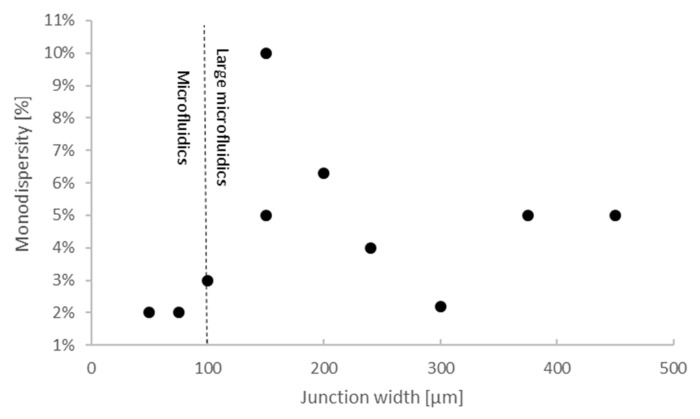
Most demonstrated 3DP droplet microfluidics were able to generate droplets with monodispersities lower than 6%. Comparing the junction width and the reported monodispersities, we can see that most 3DP droplet generators were capable of generating droplets with ~5–6% monodispersities on average in the large microfluidics size domain. In the microfluidics size domain below a 100 µm junction width, a monodispersity better than 3% was also demonstrated.

Next, we analyze the relation between the junction width and the generation rates (Figure 5). Most papers that reported generation rates had chips in a 100–500 µm channel dimension. Out of these, most had <100 Hz for the maximum generation rate [45,109,110,113]. However, in one case, the reported generation rate was 27 kHz [79]. All except one of these papers reported 5–6% monodispersity (Figure 6). Therefore, we can conclude that a throughput of up to 100 Hz is realistic and currently achievable with 3DP droplet generators. With the option to parallelize channels or chips, this rate can be increased significantly.

To summarize, in this analysis, we showed that 3DP droplet generators are viable with certain restrictions. Most chips are functional in the large microfluidics size domain of 100–500 µm for the channel dimension. They can generate droplets with diameters between 50 and 500 µm, with 5–6% monodispersity and up to a 100 Hz generation rate. The generation rates and droplet diameters are comparable to the lower end of PDMS microfluidics, but the monodispersity is still lacking in comparison.

### 2.3. Applications of 3D-Printed Droplet Microfluidics

In the previous section, we looked at the technology used for 3D printing droplet microfluidic chips and their droplet generation performance. To further assess their viability on a larger scale, we must analyze biological and chemical applications using 3DP droplet microfluidic chips. We will not go into details on applications demonstrated in particular papers, as that has already been done in several reviews in the past [6,18,19,20,21,22]. For the analysis in this section, we used the same dataset as in Section 2.2.

First, we must quantify and classify the demonstrated setups in the surveyed papers. The categories used for classification will be the following:

- **Fluid mechanics** [38,44,45,51,66,79,92,94,102,107,111,114,115,116,117,118], namely papers on fluid manipulation not involving any biological or chemical application. This also includes papers about droplet generation performed by itself without any further application of droplets;
- **Entrapment** [39,91,113], or immobilizing microparticles (e.g., cells and proteins). Commonly, this involves using a lattice or micropillar array, microwells or similar structures. The category can also include other means of immobilization (e.g., immobilization with chemical or electromagnetic means);
- **Chemical analysis** [40,93,95,101], particularly quantitative or qualitative analysis of chemicals in a sample, usually a flow chemistry application where a specific molecule is detected;
- **Diagnostics** [62,109,110], as in the detection of biological materials, be it bacteria, viruses or other microorganisms (e.g., E. coli). The category also includes the detection of nucleic acids and proteins. This category is closely linked with potential point-of-care applications;
- **Bioanalysis**, covering the implementation of bioanalytical techniques such as gel electrophoresis or viscometry in an integrated format. This also includes the biomonitoring of liquid samples (e.g., impedimetric flow cells);
- **Cell culture**, as in cell culturing applications. In this category, the focus is on sustaining cells, not necessarily monitoring their activity or response to stimuli;
- **Chemical synthesis** [68], such as pharmaceuticals. This category also includes nanoparticle synthesis;
- **Biocompatibility analysis**, analyzing the biocompatibility of 3DP microfluidics;
- **Special or other**, which includes works that do not fall into any other category (e.g., implementing fluidic logic circuits).

**Figure 7:**
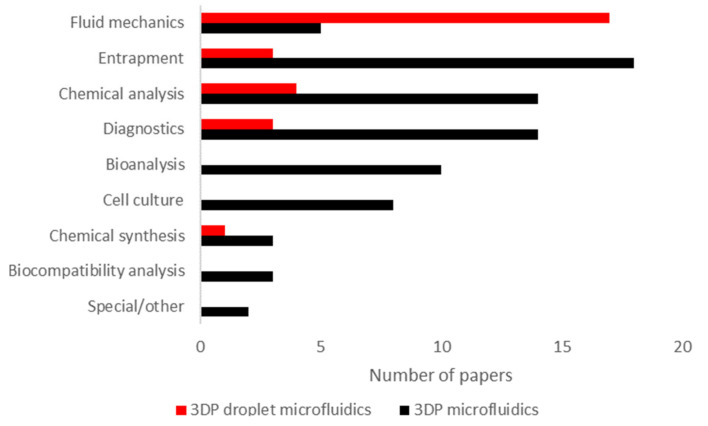
In the last five years, most 3DP droplet microfluidics applications were limited to fluid mechanics, whereas overall 3DP microfluidics saw the most applications in the entrapment of microparticles. Application categories are defined in detail the text. From a total of 106 surveyed 3DP microfluidics papers (for detailed information, please see Table S2), 30 were specific to droplet microfluidics. Most of these only focused on basic fluid mechanics, including droplet generation and manipulation. This shows that the field is still at an early stage. Out of all the papers, the most popular application areas of 3DP microfluidics were microparticle entrapment, chemical analysis and diagnostics.

Figure 7 shows the distribution of papers in the categories described above. We also compared the distribution for all 3DP microfluidics papers in general, as well as 3DP droplet microfluidics specifically. For 3DP microfluidics in general, by far the most popular application area is the entrapment of microparticles [39,41,50,55,56,57,61,70,75,89,91,96,99,100,104,113,119,120]. Chemical analysis and diagnostics are also highly popular [42,47,49,52,53,59,67,77,86,90,97,103,106,121,122,123,124,125,126,127,128,129,130]. In comparison, for droplet microfluidics, the most popular application area is fluid mechanics. This is likely due to the fact that the basic technology has not yet been established well enough to have an abundance of more complex applications. Most 3DP droplet microfluidics papers in the last five years have focused on solving the problem of droplet generation [44,69,92,102,107,108,111,112,114,115,116,118]. In the next five years, we will likely see more papers on biological or chemical applications as 3DP microfluidic droplet generation becomes an everyday practice.

## 3. Democratizing 3DP Droplet Microfluidics

It is clear from the number of publications (Figure 1) that PDMS is still the most popular structural material for both continuous flow and discrete flow applications. While PDMS has excellent qualities for microfluidic applications, it also has associated challenges and disadvantages [9,131]. In particular, soft lithography (1) is time-consuming, (2) often has poor repeatability of fabrication, (3) has expensive chip design changes, (4) is difficult to automate and (5) does not have standardized mold design formats, limiting design transfer and automation. These disadvantages limit the fabrication of PDMS chips to small quantities.

In contrast, 3D printing (1) is highly automated, (2) has design exchange file formats (STP and STL) are standardized and (3) has a short learning curve. Therefore, 3D printing could be an ideal candidate for fabricating plastic microfluidic chips with a low entry barrier [1,11].

3D printing can make testing new microfluidic chip designs easy, but as we showed in the previous section, 3DP chips could also be used for biological or chemical analysis directly. 3DP chip fabrication is also less labor-intensive and time-consuming than PDMS prototype fabrication. Therefore, in an ideal future, every microfluidics lab should have their own 3D printer for fabricating chips on a daily basis. Furthermore, it would be essential to have user-friendly means for designing and quickly evaluating new microfluidic channel layouts. How do we get there? This is what we will analyze in this section. Beyond that, we will analyze how 3D printing and novel digital automation tools compatible with 3D printing can supplement or improve traditional microfluidic fabrication workflows both in research and in the industry.

### 3.1. Increasing Availability in Mainstream Laboratories

Entry-level FDM and SLA printers are available for under EUR 500, including a small supply of printing material. For instance, the Prusa Mini FDM (EUR 419) [132] and Anycubic Photon SLA (EUR 228) [133] printers require minimal to no assembly by the user, yet they have excellent software and community support as well as training materials and configuration guides. A kilogram of premium filament for FDM printers costs EUR ~30, but basic filament spools can start as low as EUR 10/kg. SLA resin is slightly more expensive at EUR ~50–60/liter. However, the material required to print a standard microfluidic chip of a 25 mm x 75 mm x 1.5 mm envelope is ~4 g of filament for FDM and ~3 mL of resin for SLA (estimation by a PrusaSlicer from Prusa Research, Prague, Czechia [134]). Therefore, the cost of a single chip can be as low as EUR 0.15 for SLA and EUR 0.12 for FDM, considering material costs. Even on entry-level printers, up to four copies of the same chip will easily fit on the platform and can be printed in one go in ~0.5 h with FDM and ~2.5 h with SLA. Furthermore, if all chips are the same height, the printing time for SLA will not increase when adding more copies of the same chip, as the printing time is determined by the number of layers printed. The tools and materials needed to use printers (e.g., gloves and isopropanol for SLA) are available in most research labs or are supplied with the printer.

However, easily accessible and usable chip designs are also necessary to increase the availability of printers. Today, open-source, community-driven 3D printing repositories are available on the Internet, such as Thingiverse [135], which enable users to download and print designs without any knowledge of computer-aided design (CAD) software or designing 3D parts. Recently, similar repositories and libraries have been proposed and built for microfluidic chips [2,3,14,22,32]. One repository that stands out is Metafluidics [136], which is conceptually similar to Thingiverse and was started by the MIT Lincoln Laboratory. Open-source design repositories enable community-driven creation and exchange of chip designs, as well as design transfer between physically distant labs, further decreasing the entry barrier to 3DP microfluidics. In a future where 3DP microfluidics is more widespread, researchers could search for premade and tested designs for their particular research problem, then download and print chip designs on their own systems.

However, to maximize the potential of this field, standardization of chip formats and interfaces would be essential (as is generally the case for all microfluidics). Having standardized chip libraries would enable the mixing and matching of various chips to create the desired flow path with minimal engineering knowledge. On the other hand, online repositories need not be fully open source either. For-profit design libraries could also offer professional chip designs at a price point that is still far more affordable than buying the chips themselves. Since no logistics or fabrication are involved, fabless microfluidic chip design companies could operate with far less equipment and a smaller staff. At present, one typically buys chips from a catalog and waits for delivery. Instead, researchers being able to purchase designs from online catalogs and print them in their own labs would eliminate delays completely. Besides chips, specialized infrastructure could also be printed on the spot (e.g., chip holders, fittings, frames and stands). There is another challenge related to standardization which must be addressed: tolerances, and linked to that, repeatability. Due to vendor fragmentation and no industry-wide standardization, each lab will have unique tolerances (per printer, materials used, printing methods and ambient conditions among other factors), which currently have to be established in every lab individually. At present, this challenge is also largely unaddressed in the literature.

In summation, considering printers and materials, most labs today could already afford to have at least one printer. Recent progress related to readily available chip designs is promising, but more work is needed for the standardization of chip formats and interfaces. The number of repositories should increase, and the field should attract the attention of private companies dedicated to making professional designs. With these needs addressed, far more labs could gain access to 3DP microfluidics, up to the point where every lab could have one. In the future, cloud-based manufacturing using 3D printing could potentially trivialize microfluidic chip fabrication in research. This would allow researchers to focus more on biological and chemical applications rather than basic infrastructure.

### 3.2. Digital Design Automation and Future Potential

Digital twins are a fairly novel concept brought on by recent developments in Industry 4.0 [137]. Essentially, digital twins are virtual representations of physical systems that behave and respond to stimuli like the physical system. This is realized by constructing virtual models and feeding them with sensor data from physical systems. In the case of microfluidics, the model can be implemented in finite element modeling (FEM) and fed with experimental data to represent real-life systems in operation. This model can then be used for the extrapolation or interpolation of parameters under various operating conditions or to test minor design changes. Several examples in the literature have utilized computational fluid dynamics (CFD) or finite element modeling (FEM) simulations to analyze various aspects of their microfluidics devices. Commonly, simulations are used to evaluate the effects of microchannel geometry changes on flow conditions [67,88,98,138,139,140,141]. They can also be used to analyze the effect of channel geometry on reaction processes [47,142]. However, they can also be used to study electromagnetic or thermal effects. For instance, they can be used to analyze heat distribution under various operating conditions in microchannels where measurements would be difficult in situ [143,144]. Furthermore, they can be used to analyze electromagnetic fields and optimize electrode or magnet placement [100,145]. The basics of digital design and the simulation of microfluidics are covered in the preexisting literature [146,147]. Therefore, we will not go into details on these topics in our description and will only focus on the benefits to 3DP microfluidics in general and 3DP droplet microfluidics in particular.

**Figure 8:**
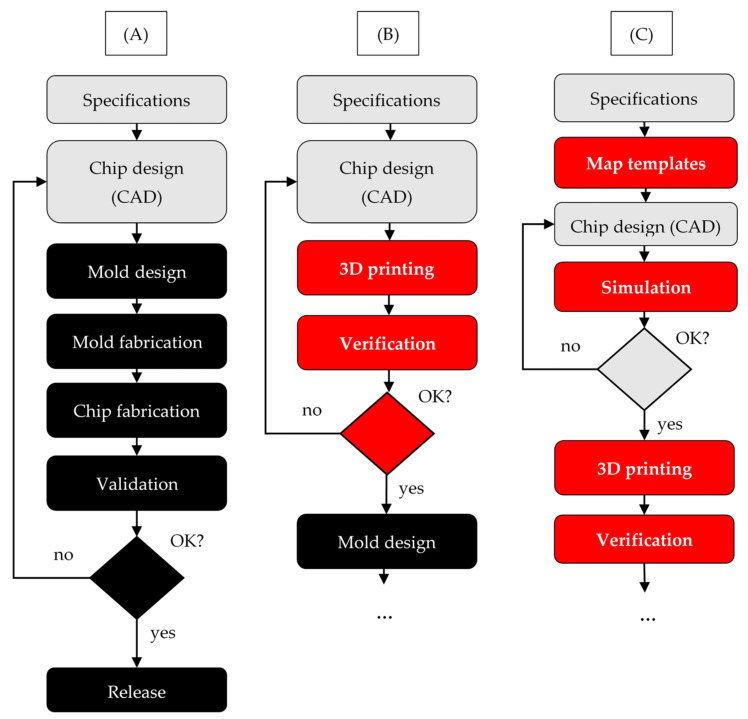
Combining cloud-based and online design sharing, digital twins and 3D printing could greatly decrease microfluidic chip fabrication costs and time. The microfluidic chip design (gray) and production (black) workflows are illustrated. Process steps related to digital twins, online design sharing and 3D printing are marked in red. In a traditional workflow (**A**), chip design is immediately followed by mold and chip fabrication, be it for PDMS or injection molding. If the design validation fails, a redesign incurs significant costs. In an alternative workflow (**B**), 3D printing is used to pre-test chip designs, and only after this test is successful is the mold designed and fabricated. However, to realize the full potential of 3D printing (**C**), digital twins should be used in conjunction with 3DP designs to speed up the process and save even more on fabrication costs and time. Furthermore, by using design templates from online repositories, the chip design process can be sped up and simplified. After this, the workflow can continue with mold design and fabrication. In the future, the end-user device could also be 3DP, depending on the use case.

Combined with 3D printing, FEM simulations can be powerful tools that can greatly speed up prototyping and design evolution. In a traditional PDMS–glass microfluidic chip design workflow (Figure 8A), expensive and time-consuming processes such as mold fabrication and chip fabrication must precede design validation and design release. If a design error is made or a new design is needed, molds have to be replaced. Instead, 3DP chips could be used to verify chip designs before the soft lithography mold is designed (Figure 8B). This verification can involve solely flow mechanics testing (e.g., pressure measurements) or testing with a biological or chemical assay [144]. Only after the 3DP design verification should the process continue with mold design, according to Figure 8A. However, to realize the full potential of 3D printing and digital design automation, we propose a third approach (Figure 8C). As mentioned in Section 3.1, design repositories for microfluidic chips are being created. These designs could be used as templates and mapped to desired chip specifications to speed up the design process. Then, CFD or FEM simulations could be used to evaluate the design before it is 3DP and tested for the first time. If the simulation does not yield expected results, the design can be changed. This in silico design evolution cycle can save both time and costs. After 3D printing, there are two possible paths. Either the process continues with the traditional workflow according to Figure 8A, ending in a PDMS–glass end-user device, or the end-user device could itself be 3DP. As we have shown in Section 2, 3DP droplet microfluidics are capable of functioning as both droplet generators and hosting biological or chemical assays. Therefore, in the future, 3DP devices may be used in clinical or research laboratories. We will analyze this possibility further in the next paragraph.

What if end-user devices were 3DP? At present, point-of-care devices, including microfluidics, are mass-produced with injection molding [148,149]. An ideal application of 3DP microfluidics is the small-scale, localized production of parts, such as in cities for distribution to local research or clinical laboratories. Considering the standard chip size, SLA printing and the printing time mentioned in Section 3.1, about 40 chips can be printed per day per printer if operated 24 h a day, meaning that even a small printer farm with three printers could produce up to 100 chips per day, and 30 printers can produce over 1000 chips per day. Therefore, in theory, large-scale production on 3D printers is possible, but mass production (100,000–1,000,000 parts per annum) is cheaper and more robust in factories that perform microinjection molding [17]. The quality of injection-molded parts is also superior to 3DP parts, and the material selection is wider. However, small-scale printing of up to 100 chips per day could be performed mostly autonomously on a small printer farm, where operators would only be needed to maintain the printers, refill the materials and remove completed parts. Uploading designs and monitoring printers could be done remotely. Compared with PDMS technology or injection molding, the key advantages to using 3D printing in a microfluidic chip production workflow are as follows:

- Reduced logistics. Since only 3D printing material needs to be supplied and stocked, logistics costs, overhead and environmental impacts are significantly reduced. Furthermore, new designs can be transferred digitally and do not require transporting physical masters, as would be the case for PDMS, or molds, as would be the case for injection molding;
- Design flexibility. There are no dedicated molds or masters, and therefore design changes can be quickly implemented, regardless of the number of printers involved or the number of parts printed;
- Automation. Printers can run autonomously and can be monitored remotely. In the case of SLA printers, washing and post-curing stations are also offered that can automatically finish fabricating the part. The operator only needs to move parts between machines and remove the supports at the end.

To summarize, digital design automation (the use of digital twins in particular), online design repositories and in silico design verification via simulation could greatly speed up microfluidic chip design as well as reduce the associated costs. 3DP droplet microfluidics are possible to use for running biological or chemical reactions, and their fabrication can be automated more easily than PDMS–glass chips. Small-scale chip production is possible with minimal investment and workload. Furthermore, they offer better design flexibility, as the geometry can be changed any time without having to produce new molds.

## 4. Discussion

To return to the question we asked in the introduction, is it time for every research lab to own a 3D printer? Based on our analysis in Section 2 and Section 3, 3DP microfluidic chips are adequate for rapid prototyping purposes, where a fast turnaround time is needed. They are also capable of housing chemical and biological assays. Entry-level 3D printers are highly affordable and easy to use. For microfluidics experts, open-source online repositories such as Metafluidics enable downloading and printing chip designs without extensive knowledge of CAD software. Therefore, depending on individual needs, the answer is yes. If fabrication times, automation and microfluidic chip costs are important factors in a lab that often changes chip designs, then a 3D printer can be an essential tool.

Can 3D printing bring droplet microfluidics to every lab? The answer to this question depends on the application of the droplet microfluidics chips. Most designs presented to date have been applied only as droplet generators and manipulators. In addition, most existing 3DP droplet generators were demonstrated in the 100–500 µm channel dimension range, which is possible with most SLA printers. These droplet generators can safely generate droplets at up to a 100 Hz generation rate and with a monodispersity lower than 6%. While most labs could potentially afford to buy an entry-level SLA 3D printer, the question is rather whether the chips it can currently print are adequate for the research tasks at hand. If a group is just starting out in the field of droplet microfluidics, then there is no faster way to get chips ready, and they will certainly be capable of generating droplets. However, it may be another five years before 3DP droplet generators can be widely applied in biological or chemical analysis. Based on the papers published in the last five years, it is clear that 3DP droplet microfluidics has just started, and the growth rate is close to exponential. If in the last five years we have seen development constrained to basic fluid mechanics, surely in the next five we will see significantly more results in biological or chemical applications. Therefore, it may not yet be the right time to apply 3DP droplet microfluidics in a professional setting. However, it is the ideal time to join research on it.

In general, the same can be said about 3DP microfluidics in general. It is the right time to join the effort, as the popularity of PDMS compared with 3D printing has dropped significantly in the last five years, since 3D printing offers unique advantages, enabling digital automation of both design and fabrication, freeing up personnel and saving costs. Biological and chemical applications are more numerous for 3DP microfluidics overall, and entrapment of microparticles in particular has been a popular field recently. We predict that more labs will adopt 3D printing microfluidics within the next five years, as printer and material costs are dropping. Biological and chemical compatibility is becoming less of a concern as more specialized materials are being developed. The two most popular printing methods, FDM and SLA, are both capable of producing standard-size microfluidic chips for less than EUR 0.2 and in less than 3 h. Additionally, even entry-level printers can print four copies in a single process. To maximize the potential of 3D printing, digital design automation should involve design reuse from online repositories as well as digital twins. Through simulations, design verification need not be performed on physical parts, but when ready, physical parts could be quickly made for testing with 3D printing. More traditional means of fabrication, such as PDMS, soft lithography or injection molding, should only come at the end of the design process, after the design is already verified in silico or on 3DP physical models.

Based on recent results, it seems likely that, eventually, 3DP microfluidic chips can supplement injection molding at low production numbers (up to 1000 chips). In addition, 3DP microfluidics can yield prototypes faster and with less manual labor than PDMS molding. Therefore, it may be more competitive in the future for use scenarios where a fast turnaround and automation are more important than quality. In theory, 3D printing could easily produce 1000 chips per day with minimal intervention from personnel. Furthermore, printing chips allows for design changes at any time, as no mold is involved. Therefore, in the near future, we may see small prototyping companies take orders from research labs to locally source 3DP chips of a higher quality than what is achievable with entry-level printers.

Some open challenges remain. One of the most important issues to address is standardization, which is generally poor in microfluidics in general. To achieve the same ubiquity as electronics has, microfluidics needs standardization of formats, interfaces and connectors, particularly standards that everyone can use, including people who 3D print. In addition, the monetization of design repositories should yield high quality designs that people could 3D print on their printers, eliminating most of the logistics-related costs and environmental impact related to the transportation of unique parts in low quantities. Other challenges related to environmental impact have been addressed in the previous literature, including the often-hazardous nature of resins. This is mitigated somewhat by the introduction of plant-based resins (e.g., [150]), but challenges remain. To professional users, a 100 Hz generation rate for droplet generators may be too low. In addition, the dimensional limitations associated with 3D printing may be a showstopper. The roughness and weak hydrophobicity of 3DP surfaces could pose a risk to some applications, too. Specific surface treatments to 3DP droplet microfluidics have been largely unaddressed so far. Thus, while 3DP droplet microfluidics could already potentially spread to every lab today, at the moment, technical limitations must still be considered before fully replacing more traditional means of fabrication. For quick testing and initial evaluations, SLA-printed 3DP microfluidics is already a sound choice, and so we recommend that labs buy their own SLA printers and start printing.

## 5. Conclusions

Based on this survey, we reached the following conclusive remarks:

- SLA and FDM are the best candidates for the democratization of 3DP microfluidics. Both offer the same high level of automation and flexibility; however, both have their unique technical challenges. Among other issues, SLA parts primarily have issues with biocompatibility, and FDM has issues with surface quality, those being being watertightness and printing overhangs;
- Open access hardware platforms with digital design automation, together with online design repositories and verification of designs via simulation, could greatly speed up microfluidic chip design as well as reduce the associated costs.

Considering printers and materials, today, most labs could already afford to have at least one printer. Recent progress related to readily available chip designs is promising, but more work is needed for the standardization of chip formats and interfaces, as well as establishing tolerances.

## Figures and Tables

**Table 1:** Recent review papers on 3DP microfluidics and 3DP droplet microfluidics. * = minimum one paragraph on the topic (<1 page). ** = minimum one section on the topic (~1–3 pages). *** = multiple sections on the topic (>3 pages).

Feature	Topic	Covered in Detail (***)	Covered (**)	Mentioned (*)
**Technology**	3D printing techniques (e.g., SLA, FDM), materials, chip printing	[1,2,3,4,5,6,7,8,9,10,11,12,13,14,15,29]	[20,21,22,30,31,32,33,34,35,36]	[18,19]
	Comparison of commercial 3D printers (table)	-	[1,11]	-
	Printing functional elements (actuators and sensors)	[32]	-	-
	3D-printed PDMS casts	-	[9,11]	-
	Integration with 3D printed electronics	-	[15]	[3,4]
	Technical challenges and limitations	[37]	[1,6,17,30,31]	[21]
**Applications**	Biological or chemical analysis and synthesis applications	[6,18,19,20,21,22,36]	[5,7,11,30,35]	-
	Cell cultures and organ-on-chip applications	[18,19]	-	[6,21,30]
	Bioprinting	-	[5,8,18]	[2,6]
	Biological and chemical compatibility	[16]	[2,14,17]	[1,4,11,19,31]
	Droplet microfluidics	[31]	-	[14]
**Future Potential**	Design automation tools (e.g., CAD + CFD or FEM)	-	-	-
	Digital or cloud-based manufacturing (Internet prefab libraries, modular systems)	-	[2,3,14,22,32]	[17]
	Repeatability and reproducibility of geometry or dimensions, tolerances System integration	- -	- [22]	- [31]
**Other**	Statistical analysis	-	-	[8,14]

**Table 2:** Comparison of the most widely used 3D printing techniques and key performance metrics related to printing microfluidics. Based on 106 publications in 3DP microfluidics, SLA offers the best combination of resolution, printing time and cost. FDM offers faster printing times, lower cost and better material selection at the cost of resolution. For specific numbers and paper references, please refer to supplementary material S2. In addition, examples of 3D printing materials and printers used in these papers are shown in Tables S4 and S5.

Technology Group	Technique	Channel Dimension (Typical) (µm)	Channel Dimension (Best) (µm)	Printing Time (Best) (h)	Chip Cost (EUR)
Vat Polymerization	SLA (stereolithography), including LCD (liquid crystal display) and laser-based DLP (digital light projection)	100–1000 200–500	18 [38] 150 [39]	<0.5 [40] <0.5 [39]	0.5–1
Material Extrusion	FDM (fused deposition modeling)	200–800	40 [41]	<0.5 [42]	<0.5
Material Jetting	PJM (PolyJet modeling)	500–1500	54 [43]	0.5 [44]	>1
	MJM (MultiJet modeling)	100–500	100 [45]	4 [46]	
	MJP (MultiJet printing)	200–500	200 [47]	3 [48]	

## Supplementary Materials

The following are available online at https://www.mdpi.com/2072-666X/12/3/339/s1, Table S1: Parametric searches, https://www.mdpi.com/2072-666X/12/3/339/s1, Table S2: Catalog of papers used in the review, https://www.mdpi.com/2072-666X/12/3/339/s1, Tables S3–5: 3D printers and materials.
