# Supplementary material for: Can 3D Printing Bring Droplet Microfluidics to Every Lab?—A Systematic Review
Source: Micromachines (Basel). 2021 Mar 22;12(3):339. doi: 10.3390/mi12030339 (PMC8004812; doi:10.3390/mi12030339)
Supplement: Supplementary file 1 [file micromachines-12-00339-s001.zip › micromachines-1136857-supplementary/S1_ParametricSearches.pdf]

STRING	Term	Anytime	2015-2020	2015	2016	2017	2018	2019	2020 (Q3)
**Google scholar**				**Yearly distribution**					
"Microfluidic" OR "Microfluidics"	Microfluidics (total)	635000	242600	42800	44800	47400	46200	39100	22300
("microfluidic" OR "microfluidics") AND "3D printing"	3DP (3D printed) microfluidics	18500	16460	1060	1710	2400	3430	4050	3810
("microfluidic" OR "microfluidics") AND " PDMS"	PDMS microfluidics	104000	52820	7880	8230	9000	10100	10200	7410
("microfluidics" OR "microfluidic") AND "droplet" AND "3D printing"	3DP droplet microfluidics	5910	5306	321	555	790	1130	1300	1210
("microfluidic" OR "microfluidics") AND ("injection moulding" OR "injection moulded" OR "injection molded" OR "injection moulding"))	Injection moulded microfluidics	12300	5134	675	755	786	915	993	1010
**WOS**				**Yearly distribution**					
TI=(microfluidics OR microfluidic)	Microfluidics (total)	29402	13591	2237	2229	2419	2344	2781	1581
TS=((microfluidics OR microfluidic) AND "3D print*")	3DP microfluidics	1066	1015	72	121	147	222	270	183
TS=((microfluidics OR microfluidic) AND "PDMS")	PDMS microfluidics	5911	1596	413	378	416	410	357	222
TS=((microfluidics OR microfluidic) AND "3D print*" AND "droplet")	3DP droplet microfluidics	85	83	7	7	9	19	26	15
**Scopus**				**Yearly distribution**					
TITLE-ABS-KEY (microfluidics OR microfluidic)	Microfluidics (total)	32968	18029	5717	2462	2371	2609	2743	2127
TITLE-ABS-KEY ((microfluidics OR microfluidic) AND "3D printing")	3DP microfluidics	844	795	54	102	104	183	175	177
TITLE-ABS-KEY ((microfluidics OR microfluidic) AND "PDMS")	PDMS microfluidics	6347	2211	440	389	382	390	351	259
TITLE-ABS-KEY ((microfluidics OR microfluidic) AND "3D printing" AND "droplet")	3DP droplet microfluidics	73	68	6	8	8	16	21	9

STRING	Term	Anytime	2015-2020	2015	2016	2017	2018	2019	2020 (Q3)
**Google scholar**				**Yearly distribution**					
("microfluidic" OR "microfluidics") AND "3D printing" AND ("SLA" OR "Stereolithography")	SLA	5260	4659	273	445	691	1010	1150	1090
("microfluidic" OR "microfluidics") AND "3D printing" AND ("FDM"OR "Filament Deposition Modeling")	FDM	2700	2482	140	227	364	511	637	603
("microfluidic" OR "microfluidics") AND "3D printing" AND ("SLS" OR "Selective Laser Sintering ")	SLS	2470	2097	126	199	334	463	520	455
("microfluidic" OR "microfluidics") AND "3D printing" AND ("DLP" OR "Digital Light Processing ")	DLP	1510	1420	46	94	158	300	379	443
("microfluidic" OR "microfluidics") AND "3D printing" AND ("SLM" OR "Selective Laser Melting ")	SLM	1200	1089	51	96	182	235	260	265
("microfluidic" OR "microfluidics") AND "3D printing" AND ("EBM" OR "Electronic Beam Melting ")	EBM	414	371	29	43	63	78	82	76
("microfluidic" OR "microfluidics") AND "3D printing" AND "DMLS"	DMLS	210	222	12	20	36	51	50	53
("microfluidic" OR "microfluidics") AND "3D printing" AND ("LOM" OR "Laminated object Manufacturing ")	LOM/SL	464	392	28	41	68	90	91	74
("microfluidic" OR "microfluidics") AND "3D printing" AND ("DOD")	DoD	495	398	25	41	82	77	87	86
("microfluidic" OR "microfluidics") AND "3D printing" AND ("Binder Jetting")	Binder Jetting	509	475	17	35	65	106	131	121
("microfluidic" OR "microfluidics") AND "3D printing" AND ("MJM" OR "Multi-jet OR "Multi jet")	MJM	319	283	16	28	43	69	61	66
("microfluidic" OR "microfluidics") AND "3D printing" AND ("Polyjet")	PolyJet	750	217	32	16	26	55	45	43
("microfluidic" OR "microfluidics") AND "3D printing" AND ("Ultrasonic Consolidation")	UC	72	53	3	6	6	15	14	9
("microfluidic" OR "microfluidics") AND "3D printing" AND ("Laser Engineering Net Shaping ")	LENS	7	7	1	0	2	3	0	1
("microfluidic" OR "microfluidics") AND "3D printing" AND ("EBAM " OR "Electronic Beam Additive Manufacturing")	EBAM	35	24	0	1	4	1	7	11
("microfluidic" OR "microfluidics") AND "3D printing" AND ("NanoParticle Jetting")	NPJ	10	9	0	0	0	1	4	4

STRING	Term	Anytime	2015-2020	2015	2016	2017	2018	2019	2020 (Q3)
**Google scholar**				**Yearly distribution**					
("microfluidic" OR "microfluidics") AND "3D printing" AND "droplet" AND ("SLA" OR "Stereolithography")	SLA	2047	1620	76	162	248	371	429	334
("microfluidic" OR "microfluidics") AND "3D printing" AND "droplet" AND ("FDM"OR "Filament Deposition Modeling")	FDM	996	874	37	78	120	189	221	229
("microfluidic" OR "microfluidics") AND "3D printing" AND "droplet" AND ("SLS" OR "Selective Laser Sintering ")	SLS	985	701	44	76	125	179	182	95
("microfluidic" OR "microfluidics") AND "3D printing" AND "droplet" AND ("DLP" OR "Digital Light Processing ")	DLP	623	564	13	31	58	125	131	206
("microfluidic" OR "microfluidics") AND "3D printing" AND "droplet" AND ("SLM" OR "Selective Laser Melting ")	SLM	447	383	17	34	54	54	80	144
("microfluidic" OR "microfluidics") AND "3D printing" AND "droplet" AND ("EBM" OR "Electronic Beam Melting ")	EBM	159	142	10	9	24	29	28	42
("microfluidic" OR "microfluidics") AND "3D printing" AND "droplet" AND "DMLS"	DMLS	87	71	2	5	13	14	12	25
("microfluidic" OR "microfluidics") AND "3D printing" AND "droplet" AND ("LOM" OR "Laminated Object Manufacturing ")	LOM/SL	195	152	4	22	22	39	26	39
("microfluidic" OR "microfluidics") AND "3D printing" AND "droplet" AND ("DOD")	DoD	347	276	19	25	56	57	55	64
("microfluidic" OR "microfluidics") AND "3D printing" AND "droplet" AND ("Binder Jetting")	Binder Jetting	247	222	6	14	31	55	40	76
("microfluidic" OR "microfluidics") AND "3D printing" AND "droplet" AND ("MJM" OR "Multi-jet OR "Multi jet")	MJM	176	150	6	15	25	41	28	35
("microfluidic" OR "microfluidics") AND "3D printing" AND "droplet" AND ("Polyjet")	PolyJet	289	250	4	31	41	60	62	52
("microfluidic" OR "microfluidics") AND "3D printing" AND "droplet" AND ("Ultrasonic Consolidation")	UC	37	19	1	2	2	4	4	6
("microfluidic" OR "microfluidics") AND "3D printing" AND "droplet" AND ("Laser Engineering Net Shaping ")	LENS	4	4	0	0	1	2	1	0
("microfluidic" OR "microfluidics") AND "3D printing" AND "droplet" AND ("EBAM " OR "Electronic Beam Additive Manufacturing")	EBAM	23	12	0	0	2	1	2	7
("microfluidic" OR "microfluidics") AND "3D printing" AND "droplet" AND ("NanoParticle Jetting")	NPJ	4	4	0	0	0	0	2	2
