# Supplementary material for: Can 3D Printing Bring Droplet Microfluidics to Every Lab?—A Systematic Review
Source: Micromachines (Basel). 2021 Mar 22;12(3):339. doi: 10.3390/mi12030339 (PMC8004812; doi:10.3390/mi12030339)
Supplement: Supplementary file 1 [file micromachines-12-00339-s001.zip › micromachines-1136857-supplementary/S3_3DPrinters.pdf]

Table S3: 3D printing methods

Acronym	Method	Greater grouping
SLA	Stereolithography apparatus	Vat polymerization
DLP	Digital Light Processing	Vat polymerization
LOM	Laminated Object Manufacturing	Sheet lamination
UC	Ultrasonic consolidation	Sheet lamination
SLS	Selective Laser Sintering	Powder bed fusion (polymer)
DMLS	Direct Metal Laser Sintering	Powder bed fusion (metal)
SLM	Selective Laser Melting	Powder bed fusion (metal)
EBM	Electronic Beam Melting	Powder bed fusion (metal)
DoD	Drop-on-Demand	Material jetting
MJM	Multi-jet modelling	Material jetting
PJM	PolyJet Modelling	Material jetting
NPJ	NanoParticle Jetting	Material jetting
MJP	Multijet printing	Material jetting
FDM	Fused Deposition Modeling	Material extrusion
LENS	Laser Engineering Net Shaping	Directed Energy Deposition
-	Binder Jetting	Binder jetting

Table S4: 3D Printing materials (examples from referenced papers in S2)

Acronym	Material	Type & producer
PP	Polypropylene	Generic FDM filament
PLA	Polylactic acid	Generic FDM filament
PET	Polyethylene terephtalate	Generic FDM filament
ABS	Acrylonitrile Butadiene Styrene	Generic FDM filament
PMMA	Polymethyl-methacrylate	Generic FDM filament
TPU	Thermoplastic polyurethane	Generic FDM filament
PVA	Polyvinyl alcohol (water-soluble)	Generic FDM filament
-	Somos Watershed XC 11122	DSM SLA resin
-	PIC100; HTM140; R11	EnvisionTec SLA resin
-	BV-001; BV-003; BV-007	MiiCraft SLA resin
-	VisiJet Crystal; VisiJet M3	PolyJet material
-	VeroClear; VeroWhite	PolyJet material
-	Asiga PlasCLEAR	Asiga SLA resin
-	FLGPCL02; Formlabs Clear	Formlabs SLA resin
-	FullCure 705; FullCure 720	PolyJet material
-	Accura 60	3D Systems SLA resin

Table S5: 3D Printers (examples from referenced papers in S2)

Method	Printer product	Company	Best resolution [µm]	Cost [USD]
Polyjet	Objet Eden350V	Stratasys	16	250000
FDM	Dimension Elite	Stratasys	127	50000
PolyJet	Objet Connex 350	Stratasys	16	250000
SLA	Pico+ 27	Asiga	27	12000
SLA	MiiCraft	MiiCraft	50	2300
FDM	Easy3DMaker	3Dfactories	80	2535
FDM	Replicator 2X desktop 3D printer	Makerbot	100	4000
FDM	3DTouchTM printer	Geeetech	125	3900
MJP	ProJet 3000HD	3D Systems	32	4000
FDM	Profi3Dmaker	3Dfactories	80	4665
SLA	Viper Si2	3D Systems	75	24000
SLA	Form 1	Formlabs	100	3299
PolyJet	J750	Stratasys	28	250000
MJM	Projet HD3500+	3D Systems	16	66000
FDM	ROVA 3D FDM printer	ROVA	200	2499
SLA	Form 2	Formlabs	25	3500
SLA	ILIOS 3D printer	ILIOS	12.5	3110
FDM	Ultimaker 2	Ultimaker	20	2500
FDM	SethiS3	Sethi3D	50	619
FDM	HD2X 3D Printer	Airwolf 3D	40	3995
FDM	Prusa i3 MK3	Prusa 3D	50	750
SLA	EnvisionTec Perfactory Micro	EnvisionTec	25	10000
MJP	ProJet 3510 SD	3D Systems	50	65000
